# Gelatin–Zinc Carrier as a New Method of Targeted and Controlled Release of Risedronate

**DOI:** 10.3390/ma17112473

**Published:** 2024-05-21

**Authors:** Jakub Reczkowski, Maria Długosz, Maria Ratajczak, Adam Voelkel, Mariusz Sandomierski

**Affiliations:** 1 Institute of Chemical Technology and Engineering, Poznan University of Technology, ul. Berdychowo 4, 60-965 Poznań, Poland; jakub.reczkowski@student.put.poznan.pl (J.R.); maria.dlugosz.pl@gmail.com (M.D.); adam.voelkel@put.poznan.pl (A.V.); 2 Institute of Building Engineering, Poznan University of Technology, ul. Piotrowo 5, 60-965 Poznań, Poland; maria.ratajczak@put.poznan.pl

**Keywords:** gelatin, carrier, zinc, risedronate

## Abstract

The essence of drug delivery is to use an appropriate carrier that delivers the active substance to the appropriate pathogenic site at a specific time. This study aims to develop a novel drug carrier characterized by the controlled and targeted release of risedronate (RSD). The search for new routes to deliver RSD is important because oral delivery has many disadvantages. The carrier proposed in this work is composed of gelatin, polyphosphates, and zinc. The zinc contained in the carrier is responsible for coordinating the drug. The resulting material releases RSD in a controlled manner. The rate of delivery of the substance to the body depends on the pH of the environment. This study investigated the delivery of RSD in a neutral environment, where the process exhibited a prolonged and consistent release rate. This process has also been studied in an acidic environment, which accelerates the release of the drug. Mixed-environment studies were also conducted. Initially, the drug was released in a neutral environment, and then the conditions rapidly changed to acidic. In this case, the carrier demonstrated high stability and controlled release, adapting the rate of drug release to the prevailing environmental conditions. The presented results indicate the great potential of the new gelatin-based carrier in the delivery of risedronate.

## 1. Introduction

Scientists and researchers are consistently endeavoring to develop a drug carrier characterized by precise delivery, aiming to transport active substances to diseased cells without inducing adverse effects on the human body. Due to its properties, gelatin emerges as a prime candidate for drug carrier development. As a product of collagen hydrolysis, it is a polymer of natural origin characterized by high biocompatibility, and it is also biodegradable in a physiological environment [1,2]. An additional significant advantage of gelatin is its widespread availability, primarily sourced from bovine bones, pig skin, and fish [3]. Gelatin’s functionality stems from its chemical composition, predominantly comprised of amino acids, which affords it high gel strength and a high melting point [4]. These features make gelatin widely used in drug-form technology, where it is used as a base substance in the process of producing soft capsules. The role of gelatin in this form of medicine is to prevent the oxidation of the active substance and its degradation under the influence of other factors [5]. Gelatin is found in many nanomaterials that are effective in delivering various therapeutic agents, such as small-molecule drugs, proteins, peptides, genes, etc. Its structure harbors various functional groups, offering numerous opportunities for surface modification, cross-linking, and coupling. Thanks to this, many ligands can be combined. Furthermore, this structural composition facilitates the integration of drugs or biologically active agents into gelatin’s matrix [6,7]. Gelatin is also a component of chitosan-based biocomposites, where it is responsible for improving the properties of chitosan, which has poor mechanical resistance and is insoluble in water [8].

In order to effectively use gelatin as a carrier for active substances, an appropriate linker capable of drug sorption must be used. Sodium tripolyphosphate (TPP) is a popular cross-linking agent used mainly in the food industry, where it is responsible for reducing water hardness and deflocculation in cleaning products [9]. This compound is also characterized by high biocompatibility, low toxicity, and biodegradability [10]. However, its important property is its ability to form complex bonds with metal ions, which can be used to create an appropriate drug carrier [11]. TPP combines with substances such as chitosan or gelatin using ionic bonds. This enables the formation of complex bonds with metal ions, thanks to the presence of numerous phosphate groups containing lone electron pairs. This compound forms a stable connection with zinc ions, which play an important role in modified drug delivery systems. The use of Zn^2+^ ions in drug carriers enables not only the sorption of drugs on their surface but also facilitates effective ion exchange with the components of body fluids, leading to the release of the active substance [12]. Furthermore, these ions play an important role in the functioning of the human body, contributing to the proper development of the skeletal system and supporting the process of osteointegration [13]. They also show some antimicrobial activity, therefore representing an additional advantage when creating carriers of active substances [14].

Risedronate is an active substance belonging to the group of bisphosphonates, which includes medicinal substances with a wide range of use in the treatment and prevention of bone diseases such as osteoporosis, Paget’s disease, hypercalcemia, and bone metastases [15]. The mechanism of action of this drug is based on the inhibition of osteoclasts, which are involved in bone resorption [16]. The chemical structure of risedronate allows the formation of stable complexes with metal ions based on complex bonds. According to research, risedronate forms complexes with metal cations, including Mg^2+^, Zn^2+^, Pb^2+^, and Cu^2+^ [17]. When risedronate is administered orally, the absorption of the drug is severely limited. The digestive system absorbs only a few percent (1–5%) of the administered active substance. The small amount of the drug absorbed is due to the presence of phosphate groups, which hinder the drug’s penetration through the lipid layer of cell membranes. After absorption, only half of the drug is selectively retained in the skeletal system, while the other half is excreted unchanged [18]. The most common side effects when taken orally are heartburn, osteonecrosis of the jaw, nausea, esophageal irritation, and stomach ulcers [19]. After intravenous administration of the pure drug, flu-like symptoms may occur. Another, although less common, method of administration is intranasal or transdermal administration. A side effect of these methods is local toxicity [19]. Using an appropriate carrier for this active substance could help alleviate the weaknesses of bisphosphonate drugs.

The most crucial question to address at this stage is the design of the drug carrier and its intended effect. An oral drug delivery system is unlikely to enhance the performance of the drug due to the low bioavailability of RSD. Therefore, alternative routes, such as intravenous administration and implants, are more viable options. For intravenous delivery, it is logical to develop a carrier that provides sustained release of the drug. Sustained release entails gradual dosage release, thereby reducing the drug’s intravenous toxicity. Ideally, the drug should be released from such a carrier over several hours. Moreover, an advantageous feature of such a carrier would be its ability to release the drug in response to pH reduction. This would enable higher drug doses to be released in the presence of osteoclasts, which lower the pH during bone resorption [20]. The development of drug-containing implants for osteoporosis treatment is equally important, especially considering the late detection of the disease and the frequent necessity of implant usage. These implants could take various forms, such as hydrogels containing drugs for minor defects or titanium implants coated with a drug carrier layer. Alternatively, 3D-printed titanium implants filled with a drug carrier could be used for entire bone replacements. Controlled release directly from the implant is crucial, with lower initial doses supporting osteointegration, followed by a controlled release in response to pH changes to neutralize osteoclasts and facilitate faster patient recovery.

**Figure 1:**
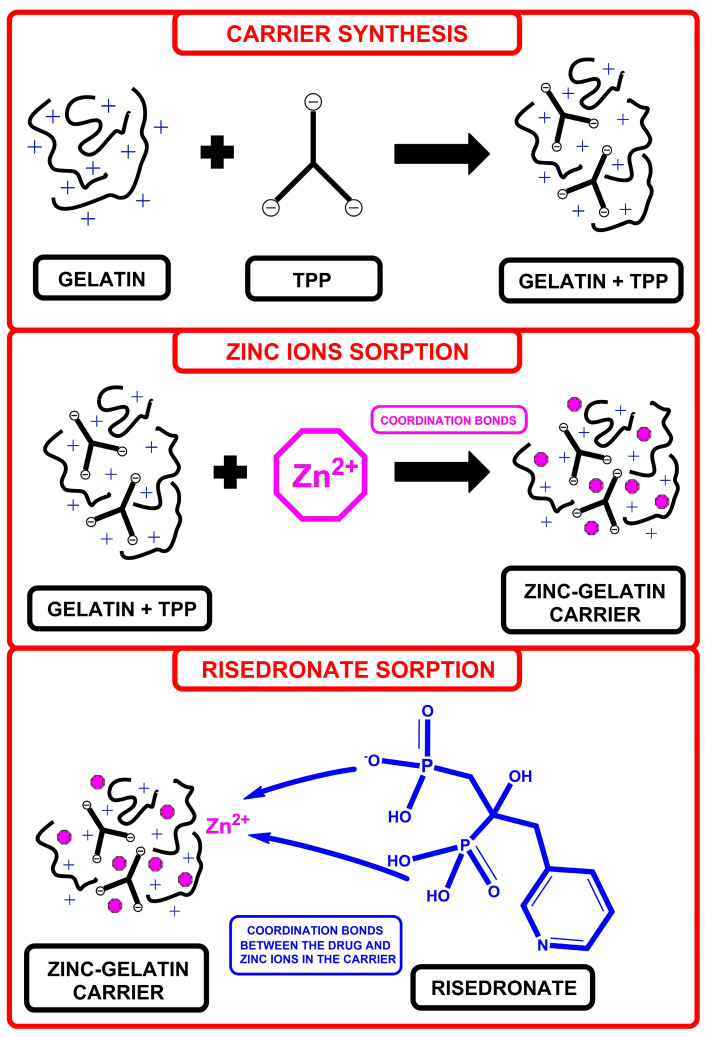
Idea and diagram of zinc–gelatin carrier synthesis.

By leveraging the structural and chemical properties of gelatin, sodium tripolyphosphate, and zinc ions, it is feasible to develop a drug carrier for risedronate characterized by high biocompatibility and biodegradability. Furthermore, the release of the drug from this newly developed carrier aims to establish a drug delivery system based on the controlled and targeted release of the active substance. The carrier introduced in this study represents just the initial phase, demonstrating potential avenues for utilizing gelatin–zinc material in intravenous or implant-based RSD delivery. The synthesis diagram and concept for the risedronate carrier are depicted in Figure 1.

## 2. Materials and Methods

### 2.1. Reagents Used for Research

The following reagents were used to synthesize the gelatin–zinc carrier: gelatin (gelatin from bovine skin, Type B, powder, BioReagent, 77% protein (Biuret), 7% water (Karl Fischer), Sigma Aldrich, Germany), sodium tripolyphosphate (>95% of purity, Sigma Aldrich), zinc nitrate hexahydrate (99% of purity, Fisher Scientific, USA), Tris(hydroxymethyl)aminomethane (TRIS, 99% of purity, Sigma Aldrich), and risedronate (99% of purity, Sigma Aldrich).

### 2.2. Gelatin–Zinc Carrier Preparation and Synthesis

**Figure 2:**
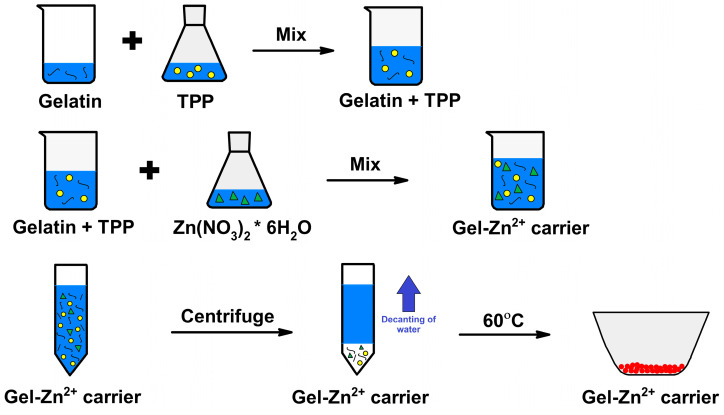
Schematic diagram of gelatin–zinc carrier synthesis.

To prepare the carrier, 200 mg of gelatin was weighed and added to 100 mL of demineralized water. In the next stage, 4000 mg of TPP dissolved in 40 mL of water was added to the previously prepared solution and then mixed until a clear solution was obtained. Subsequently, a solution of 4165.2 mg of ZnNO_3_ × 6 H_2_O in 139 mL of demineralized water was added to the solution prepared in this way. The resulting mixture was stirred, and after achieving homogeneity, it was washed and centrifuged for 5 min at 8000 rpm. The resulting product, after decanting the solution, was then dried at 60 °C. The final weight of the gelatin–zinc carrier obtained was 2132.4 mg. The material obtained after this stage of synthesis was named Gel-Zn^2+^. A schematic diagram of gelatin–zinc carrier synthesis is shown in Figure 2.

### 2.3. Drug Sorption—Risedronate

**Figure 3:**
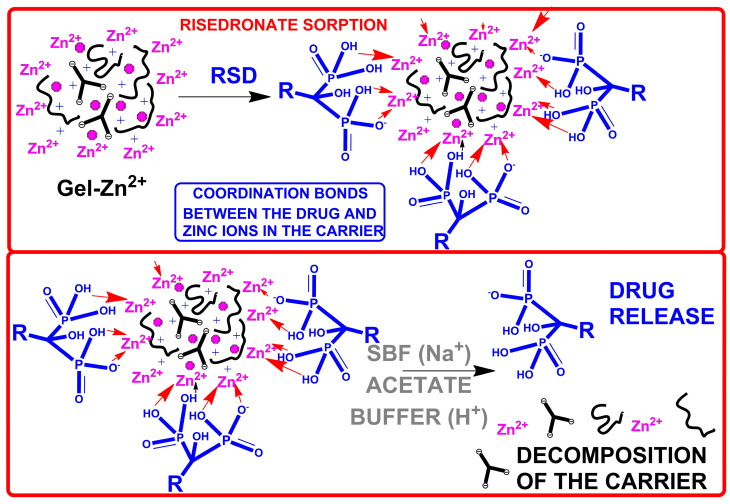
Schematic diagram of drug sorption and release.

The powder obtained in the previous stage was placed in six plastic test tubes with a capacity of 15 mL, and 20 mg of the carrier was added to each tube. Then, 15 mL of the drug solution was added to each test tube. The drug solution, with a concentration of 0.2 mg/mL, was prepared in TH buffer (TRIS-HCl). TH buffer was obtained by dissolving 0.1 M TRIS in water and adjusting the pH to 7.4 with HCl. The samples were then vortexed and placed on a laboratory rotator (Sunlab SU1010, 20 RPM) for 24 h. Following incubation, the samples were centrifuged at 4500 RPM for 5 min. The drug content in the solution was determined using UV–Vis spectroscopy. The precipitate obtained after centrifugation was dried at 60 °C for 24 h. The resulting drug-loaded carrier was designated as Gel-Zn^2+^-RSD. A schematic drug sorption diagram is shown in Figure 3.

### 2.4. Risedronate Release

The drug release process was carried out under neutral conditions, using a simulated body fluid (SBF) solution, and under acidic conditions, using an acetate buffer solution (pH 5). SBF imitated the natural conditions of the body, and the acetate buffer imitated the conditions occurring during bone resorption during the action of osteoclasts. To analyze the release of risedronate under neutral or acidic conditions, 20 mg of the prepared carrier with the adsorbed drug was weighed and placed in a test tube, where it was flooded with 5 mL of SBF solution or acetate buffer. After vortex shaking and stirring for a specified time on a rotator (20 RPM), the samples were analyzed using the UV–Vis technique. Before testing, to obtain a clear solution, the samples were centrifuged for 5 min at 4500 RPM. After centrifugation, 1 milliliter of the solution was placed in a cuvette and measured using a spectrometer. The remaining 4 mL were poured out. The carrier was then flooded with a new portion of SBF, or acetate buffer. The addition of a new portion was intended to provide a new solution to simulate conditions in the human body. Measurements for SBF were carried out every hour and for acetate buffer every 15 min. If the measured value was too small to be determined, the solution was returned to the test tube, and measurements were repeated after an extended period. The difference in measurement times results from the fact that the amount of drug in the acetate buffer was measurable after a shorter mixing time. The exact composition of the SBF solution (per 1000 mL) contained 8.035 g of NaCl, 0.355 g of NaHCO_3_, 0.225 g of KCl, 0.231 g of K_2_HPO_4_·3H_2_O, 0.072 g of Na_2_SO_4_, 0.6112 g of TRIS, and 0–5 mL of HCl. The acetate buffer was prepared according to the available literature [21]. For each condition, three measurements were performed, and the average was taken. A schematic drug release diagram is shown in Figure 3.

### 2.5. Methods

#### 2.5.1. Fourier Transform Infrared Spectroscopy (FTIR)

A Vertex70 spectrometer (Bruker Optics, Germany) was used to obtain the spectra using FTIR. When performing measurements, the reflection ATR mode was used with a diamond crystal attachment. The spectral range during the study was 4000–600 cm^−1,^ and the resolution was 4 cm^−1^.

#### 2.5.2. UV–Vis Spectroscopy

The UV–Vis spectrophotometer UV-2600 (Shimadzu, Japan) was utilized to monitor changes in risedronate concentration during sorption and release from the tested carrier. Absorption spectra were recorded in the range of 240–305 nm for each sample. In each spectrum, the maximum absorbance occurred at approximately 262 nm, a characteristic wavelength for pure risedronate. The drug concentration in all samples was analyzed at this wavelength. The amount of drug retained during sorption was determined by calculating the difference in absorbance between the initial drug solution and the solution after 24 h of sorption, using TH (0.1 M) as a background during the sorption process. For the drug release process, SBF was employed as a background in a neutral environment, while an acetate buffer was used in an acidic environment. The quantities of retained drugs were calculated based on calibration curves for risedronate in the TH solution. Similarly, the amounts of drug released were determined using calibration curves constructed for drug solutions in SBF and acetate buffer.

#### 2.5.3. Scanning Electron Microscopy (SEM)/Energy Dispersive Spectroscopy (EDS)

Images obtained using a scanning electron microscope were taken using a VEGA microscope (TESCAN, Czech Republic). The photos were prepared with magnifications of 5000 and 1000. Energy dispersive spectroscopy (EDS) was used to perform elemental analysis on the surface of the obtained carrier to check if zinc ions and risedronate were evenly distributed on its surface. The average value was obtained based on measurements at 10 points.

## 3. Results and Discussion

**Figure 4:**
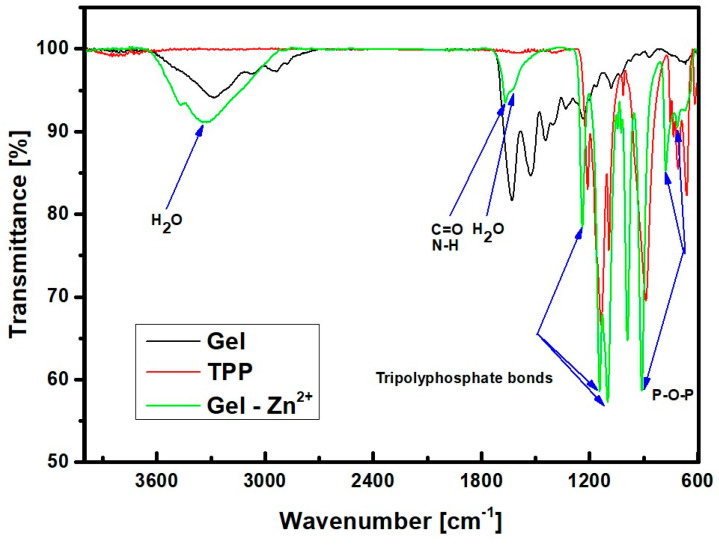
FTIR spectra of gelatin (Gel), sodium tripolyphosphate (TPP), and Gel-Zn^2+^ drug carrier.

At the outset of the study, FTIR spectroscopic analysis was employed to confirm the presence of relevant functional groups originating from gelatin, TPP, and risedronate. Initially, the spectra of the substrates (gelatin and sodium tripolyphosphate) used for the synthesis of the gelatin carrier were examined and compared with the spectra obtained for the finished drug carrier named Gel-Zn^2+^. The results are presented in Figure 4.

**Figure 5:**
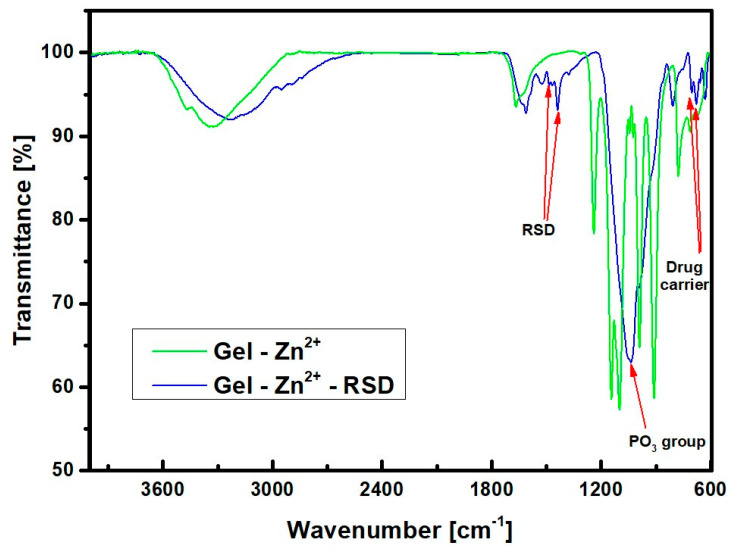
FTIR spectra of Gel-Zn^2+^ drug carriers before and after RSD sorption.

The bands observed at 3300 cm^−1^ and 1630 cm^−1^ can be attributed to stretching and bending vibrations from water molecules, respectively. The presence of groups derived from substrates in the drug carrier can be confirmed by the identification of bands associated with gelatin. Amide bands are visible in the range of 1700–1600 cm^−1^, consisting of the C=O group and the N-H group, which induce different vibration frequencies of these bands. Their weakening in the carrier structure can be interpreted as the ongoing conversion process of the amide group. This is confirmed by the shift of the peak originally at 1630 cm^−1^ (black line) to 1670 cm^−1^ (green line) in the carrier [22]. The presence of polyphosphate can be confirmed by identifying the bands 906 cm^−1^, 760 cm^−1^, and 700 cm^−1^. These lengths are characteristic of symmetric P-O-P vibrations. The 760 cm^−1^ band is the band originating from triphosphates [23]. The multiplet in the range 1180–1100 cm^−1^, as well as the band 1252 cm^−1^, comes from the tripolyphosphate bonds. In the drug carrier, all these bands are shifted by approximately 10 cm^−1^, probably due to the presence of zinc in the carrier structure [24]. The above results are similar to those presented in the literature examining a carrier with a nanoflower structure also composed of pyrophosphates and divalent ions [23]. The next set of FTIR spectra in Figure 5 shows the zinc–gelatin carrier before and after risedronate sorption.

**Figure 6:**
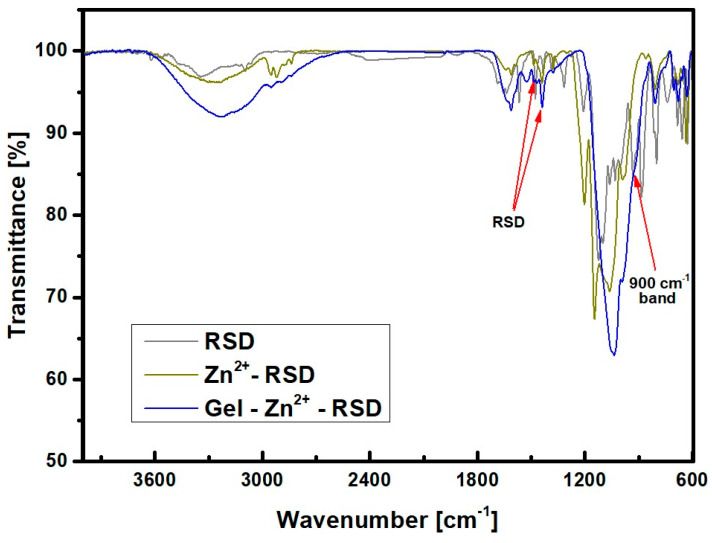
FTIR spectra comparing the ion–drug complex (Zn^2+^-RSD; drug coordinated with zinc without the addition of other substrates) with the carrier-containing drug (Gel-Zn^2+^-RSD).

This spectrum illustrates the phenomenon of band overlapping originating from the drug carrier within the range of 1700–1600 cm^−1^. The presence of the carrier is further indicated by the shifted band from 760 cm^−1^ to 800 cm^−1^. This band appears blurry due to its overlap with the drug-derived band. New bands of 1520 cm^−1^ and 1440 cm^−1^ can also be noticed. These should be attributed to the drug risedronate, which is absorbed by the zinc–gelatin carrier. The peak at 1250 cm^−1^ corresponds to the drug carrier. This band is covered because of the large amount of drug on the carrier surface. The value of 1100 cm^−1^ should be attributed to the PO_3_ group attached to the carbon bridge in the bisphosphonate structure. This is an intense band, but its blurring can be explained by the presence of a large amount of drug on the surface of the zinc–gelatin carrier. From the above spectra, it can be confirmed that the drug has been incorporated. The new bands clearly indicate the presence of RSD in the obtained material. However, it can be assumed that significant carrier decay could have occurred because the structure of the carrier–ion–drug spectra is not very close to the Gel-Zn^2+^ structure itself. This could be because the drug carrier is very abundantly covered with the drug. This obscured the carrier bands, so the drug strands are more visible than the carrier bands. This fact could have caused the formation of the ion–drug complex itself. To check whether a carrier–ion–drug (Gel-Zn^2+^-RSD) connection was formed in the sample and not an ion–drug complex (drug coordination with zinc without the presence of a carrier), additional tests were performed comparing spectra from the carrier containing the drug, the ion–drug complex, and the drug itself. The results of this analysis are presented in Figure 6.

The wavenumbers confirming the occurrence of RSD are 1520 cm^−1^ and 1440 cm^−1^, which, in combination with the zinc–gelatin carrier, exhibit a shift towards lower wavenumbers. In the sample containing the carrier, this band is slightly shifted to the right due to ion–drug interactions. The presence of a band at 900 cm^−1^ in the carrier–ion–drug sample confirms the formation of a complex between the ion and the drug. This test confirms that the drug has been absorbed in the carrier and that zinc ions have not been washed out of the solution. Slight similarities between Gel-Zn^2+^-RSD and Zn^2+^-RSD indicate that a carrier with an ion–drug complex was obtained and not the Zn^2+^-RSD complex itself, which was the intended outcome of this study.

**Figure 7:**
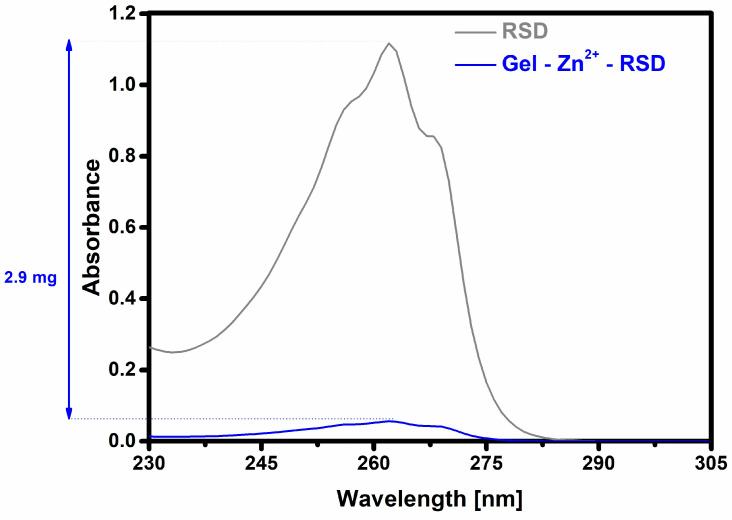
Risedronate sorption on the gelatin–zinc carrier (the difference in absorbance indicates the amount of drug retained).

In the next stage, the sorption process of risedronate was examined using UV–Vis spectrophotometry. The sorption process of this drug on the gelatin–zinc carrier lasted 24 h. The amount of retained drug was calculated based on the difference between the drug concentration in the initial solution and the drug concentration in the solution after 24 h of contact with the carrier. The results are presented in Figure 7. Figure 7 shows the characteristic UV–Vis spectrum of risedronate and the change in absorbance of the pure drug solution after adding the carrier and stirring for 24 h.

The results demonstrate that the prepared gelatin–zinc carrier was capable of sorbing 2.878 mg of risedronate, which accounts for 95.9% of the total amount of the drug present in the test solution. This value indicates that the carrier surface favors the sorption of RSD. Such a large amount of retained drug results from the formation of coordination interactions between the drug and zinc ions. The quantity of drug added was determined based on the observation that adding a larger amount caused the carrier to disintegrate, rendering it impossible to centrifuge.

**Figure 8:**
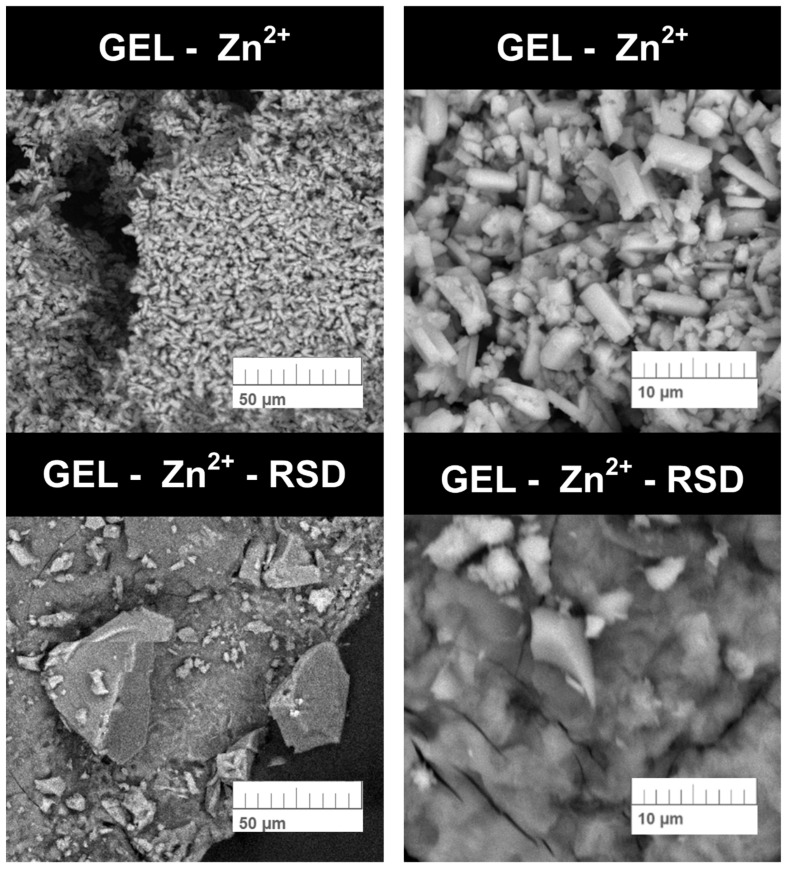
SEM photos of the obtained carrier and carrier after the RSD sorption process.

In the next stage of testing the gelatin–zinc carrier, SEM analysis was performed. The photos taken, which determine the form and shape of the obtained carrier, are shown in Figure 8.

The images of the obtained Gel-Zn^2+^ samples suggest structural similarities to zinc phosphates. However, the FTIR results (Figure 4) from this publication indicate otherwise. The spectrum of zinc phosphate previously described in the literature looks different from the spectrum presented in this work [25]. The presented particles also do not resemble zinc oxide, which indicates that another new material has been obtained, which has most likely not been described in the literature yet [26]. The photo at a lower magnification shows single crystals with specific structures that agglomerate, which may be caused by the drying process. The structures are several dozen micrometers in size, which makes intravenous administration of this carrier impossible. Due to this, it will be necessary to perform the synthesis in a different way or grind the material to reduce its particles. However, this carrier can be used on scaffolds. After drug sorption, the previously visible single crystals are no longer visible. These changes are most likely because larger crystallites disintegrated during the sorption process and the drug was retained on individual particles. This is very likely because, after pouring too much drug into the carrier, the Gel-Zn^2+^ structure disintegrated. In order to investigate the distribution of zinc ions and drug molecules on the surface of the gelatin–zinc carrier, EDS analysis was performed. The results are presented in Table 1.

**Figure 9:**
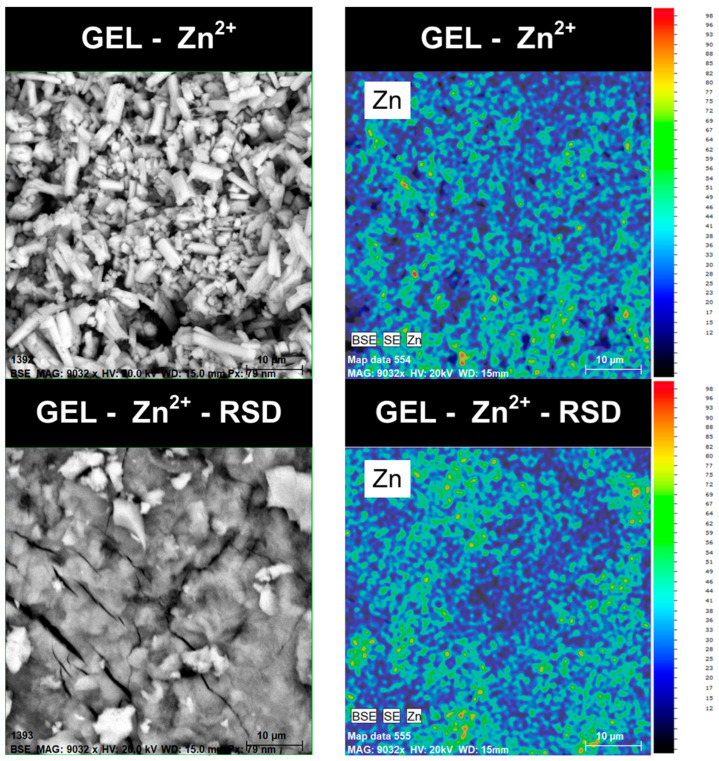
Distribution of zinc ions on the gelatin–zinc carrier surface based on EDS mapping.

The presence of the aforementioned elements indicates the presence of gelatin, TPP, and zinc ions, as well as the presence of RSD on the surface of the tested carrier. The significant increase in carbon content (up to 50.51%) after the sorption process is attributed to the abundant presence of this element in the RSD molecule, further confirming the effective sorption of the drug. The reduction in the amount of zinc is caused by the fact that RSD molecules effectively form coordination bonds with these ions, reducing the amount of free cations of this metal on the surface of the carrier. The distribution of the zinc ions is shown in Figure 9.

The obtained photos show that zinc ions are evenly distributed over the entire surface of the sample both before and after RSD sorption. This proves that the carrier is well synthesized before the drug sorption process and that the carrier structure is durable after sorption. Both after the synthesis of the carrier and after the drug sorption process, no zinc agglomerates are visible, which indicates that the material is most likely homogeneous. The presence of agglomerates could indicate that crystallites are present in the structure of the gelatin–zinc carrier.

**Figure 10:**
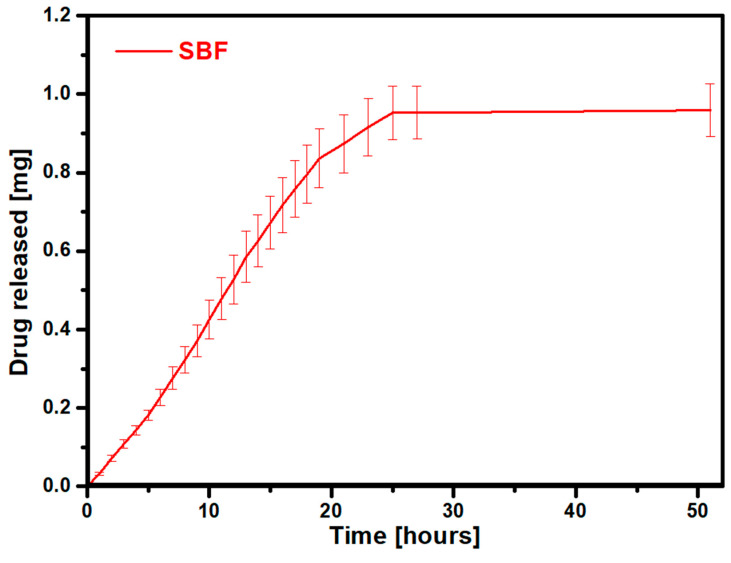
Risedronate release profile in SBF.

The next stage of the research involved analyzing the release profile of risedronate after confirming its sorption in the gelatin–zinc carrier. The release process in a neutral environment lasted 25 h. The amount of drug released was 0.96 mg, which is 33.3% of the adsorbed amount in the gelatin–zinc carrier. The release of the analyzed drug, risedronate, in the treatment of osteoporosis should be as long as possible, which would enable a long-term period of constant delivery of the drug to the site of the disease. This material can be used in the treatment of osteoporosis because, in diseased areas, conditions are favorable (not acidic) for the release of the drug for a very long time in small doses. Conditions around the implant only turn acidic when there are large numbers of osteoclasts, but in this case, a rapid release of a drug that kills the osteoclasts is desirable. This fact allows the use of this material, for example, in scaffolds, on the surface of titanium alloys, in titanium pores, etc. The release profile of the tested drug is presented in Figure 10.

**Figure 11:**
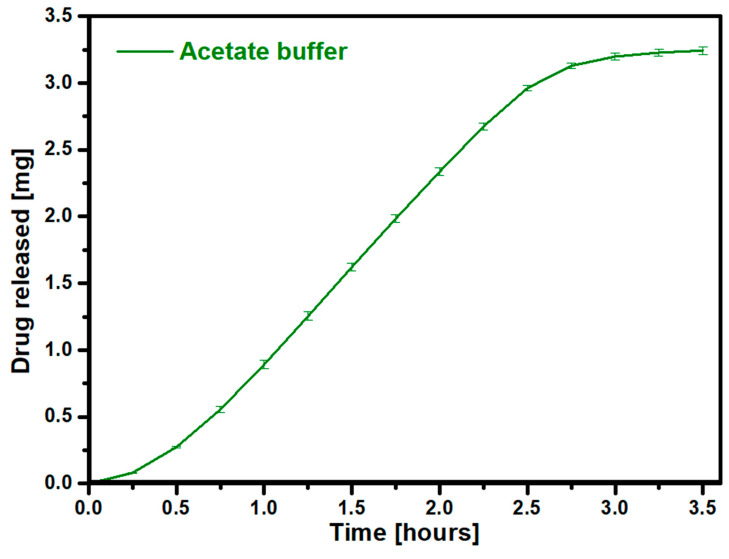
Risedronate release profile in acetate buffer.

The presented results show that the release process remains stable for approximately 25 h, indicating the potential for uniform and sustained drug delivery to the disease site over an extended period. Importantly, as the drug was released from the carrier, the carrier itself underwent progressive disintegration. It can be concluded that such a situation would prevent the formation of insoluble complexes in the body. It also makes it possible to complete the breakdown of the carrier and delivery of its substrates to the body, which supports the absorption of the drug. Additionally, the influence of acidic conditions generated by osteoclasts on drug release was investigated during the studies. The release process lasted 210 min, and the results are shown in Figure 11.

**Figure 12:**
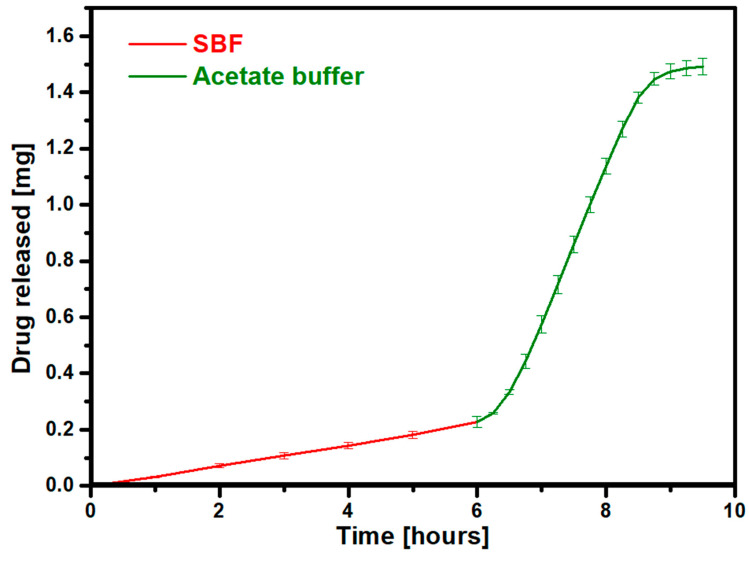
Risedronate release profile in variable conditions.

The release of the entire drug in a very short time in an acidic environment is very important in the treatment of osteoporosis. The sudden administration of a large dose of the drug can significantly disrupt the functioning and progression of the disease by preventing the growth of osteoclasts. The above requirements are met by the prepared gelatin–zinc drug carrier with adsorbed RSD. Rapid release in response to pH is desirable for implants that are disassembled by osteoclasts at an acidic pH. The release of the drug will reduce the number of osteoclasts. The last performed release analysis was to determine the release profile of risedronate under variable pH conditions. For this purpose, the process was initially carried out under neutral conditions using SBF and then changed to acidic conditions using an acetate buffer. The results are presented in Figure 12.

The results indicate that the drug is released to a greater extent when the environment is changed to an acidic one. Initially, the drug desorbs slowly and gradually upon exposure to the acidic environment, but there is a sudden increase in the release rate afterward. This change in the amount of drugs released under the influence of pH is required for the delivery of bisphosphonates. The presented results are very favorable because they prove that a change in the environment does not stop the release process or completely degrade the drug but accelerates the release of the active substance as it should in an acidic environment. This result shows that regardless of the environment, the drug will be released in the body along with ions that are intended to support the action of the drug. This is targeted drug release because, in intravenous delivery, the active substance will initially release smaller doses at a neutral pH, and only on contact with a lower pH will large doses of the drug be released.

## 4. Conclusions

In this study, we successfully developed a drug carrier capable of targeting and controlling the release of risedronate. The synthesized gelatin–zinc carrier demonstrates sustained release of the active substance, rendering it suitable for osteoporosis treatment where consistent drug delivery to the therapeutic target is vital. While the prepared carrier with the loaded drug holds promise for use in implants, further research is needed to optimize its application for this purpose. Embedding the material in a polymer matrix, such as within the pores of 3D-printed titanium, could prolong drug release. However, the current form of the material is unsuitable for intravenous injection due to its large particle size. Further investigation is warranted to reduce the particle size to nanoparticles for intravenous administration. Controlled release in response to pH reduction is particularly crucial in osteoporosis treatment, as osteoclasts create an acidic microenvironment where higher drug concentrations are required. Notably, the material containing absorbed RSD is unstable at a neutral pH, leading to its dissolution. While this may compromise carrier stability, it prevents the formation of insoluble complexes in the body, thereby minimizing the risk of undesirable side effects associated with carrier degradation. Overall, the results presented in this study highlight the promising potential of the new material in drug delivery applications.

## Figures and Tables

**Table 1:** The number of elements on the surface of the gelatin–zinc carrier [atomic%].

	Gel-Zn^2+^	Gel-Zn^2+^-RSD
C	13.96 ± 3.43	53.06 ± 3.14
O	37.82 ± 14.57	33.23 ± 4.53
Zn	20.05 ± 5.94	7.73 ± 2.19
P	28.17 ± 7.63	5.98 ± 1.58

## Data Availability

The original data presented in the study are openly available in RepOD at https://doi.org/10.18150/DTZNQ9.
